# Effects of Bu Shen Yi Sui Capsule on Th17/Treg cytokines in C57BL/6 mice with experimental autoimmune encephalomyelitis

**DOI:** 10.1186/s12906-015-0572-0

**Published:** 2015-03-12

**Authors:** Qi Zheng, Tao Yang, Ling Fang, Lei Liu, Haolong Liu, Hui Zhao, Yiyi Zhao, Hongzhu Guo, Yongping Fan, Lei Wang

**Affiliations:** School of Traditional Chinese Medicine, Capital Medical University, Beijing, 100069 PR China; Beijing Tian Tan Hospital, Capital Medical University, Beijing, 100050 PR China; Beijing Institute for Drug Control, Beijing, 100035 PR China

**Keywords:** Experimental autoimmune encephalomyelitis, Multiple sclerosis, Bu Shen Yi Sui Capsules, Th17, Treg, Interleukin-17A, Fork head box P3

## Abstract

**Background:**

T helper (Th) 17 and regulatory T (Treg) cells play a critical role in the pathogenesis of multiple sclerosis (MS) disease. Bu Shen Yi Sui Capsule (BSYSC), a traditional Chinese medicine formula, has been used clinically for the treatment of MS patients in China.

**Methods:**

To evaluate the neuroprotective effects and the underlying mechanisms of BSYSC on MS, experimental autoimmune encephalomyelitis (EAE) model in C57BL/6 mice was induced with myelin oligodendrocyte glycoprotein (MOG) _35–55_. Th17 and Treg cells and the related cytokines were detected by flow cytometry, ELISA, real-time quantitative reverse transcription PCR, western blot and immunohistochemistry.

**Results:**

We found that BSYSC improved neurological function, reduced inflammatory cell infiltration and damage to the axons and myelin in the brain and spinal cord. BSYSC down-regulated markedly the ratio of CD4 + IL-17+/CD4 + CD25 + FoxP3+ T cells in the spleen, decreased the cytokines of IL-17A, IL-6, IL-23, TGF-beta1 in the brain, and dropped the ratio of IL-17A and FoxP3 mRNA and protein in the brain or spinal cord at different stages.

**Conclusions:**

The study demonstrated that BSYSC had a strong neuroprotective effect on EAE mice. The protective mechanisms of BSYSC might be associated with mediating the regulation of Th17/Treg cells.

## Background

Multiple sclerosis (MS) as an inflammatory demyelination disease in the central nervous system (CNS) is pathologically characterized by multifocal inflammation, demyelination, and neuronal damage [1]. Experimental autoimmune encephalomyelitis (EAE), has similar pathological and clinical manifestations to human MS, is the optimal animal model of this disease [2].

A large number of studies have shown the autoimmune responses of MS or EAE are caused mainly by CD4+ T cells and the generation of a T helper (Th) 1/Th2 imbalance [3]. Recent researches have indicated the Th17/regulatory T (Treg) imbalance is a more critical factor in the mechanism underlying the neurological dysfunction of MS/EAE [4]. Th17 cells are a subpopulation of CD4+ T cells that mediate inflammatory responses, while Treg cells are a type of negative immune regulatory cells that mediate immune tolerance. The interactions between Th17 and Treg cells are regulated by the secretion of numbers of cytokines such as interleukin (IL)-17 and transforming growth factor (TGF)-β1, and specific transcription factors, including retinoic acid-related orphan receptor (ROR) γt and forkhead box P3 (FoxP3) [5-7]. The pro-inflammatory cytokines attack the myelin and axon, consequently, demyelination and axonal injury occur and result in the onset of MS/EAE [8].

Current therapeutic medicines for MS include corticosteroids and immunosuppressors, such as interferon, glatiramer acetate and natalizumab, show positive effects in inhibiting inflammation since symptoms onset [9]. Thus, prednisone acetate (PA) was selected as the control drug in the present study. Nevertheless, these therapies have potentially side-effects [1]. Traditional Chinese medicine (TCM) can be used to treat the complex and varied presentations of MS, with few side-effects [10]. Bu Shen Yi Sui Capsule (BSYSC, originally named Erhuang Capsule) is a yin-nourishing, phlegm-resolving and blood-activating formula of TCM. The previous clinical studies showed BSYSC had the ability to markedly reduce and eliminate symptoms such as limbs weakness and paresthesia, reduce the frequency and intensity of relapses, ameliorate side-effects of PA and reduces the dose of medication required, to improve the qualities of life in MS patients [11,12]. And it has been approved by the Beijing Food and Drug Administration as a hospital preparation (No. 10003).

Our previous studies have demonstrated BSYSC can regulate Interferon (IFN) -γ and IL-4 production to balance Th1 and Th2 in the serum, brain and spinal cord of EAE rats [13-15]. The aim of this study was to explore the regulatory effects of BSYSC on Th17 and Treg cells. Results showed BSYSC had a strong neuroprotective effect on EAE mice, which might be associated with the regulation of Th17/Treg cells.

## Methods

### Medicines and reagents

PA was purchased from Zhejiang Xianju Pharmaceutical Co., Ltd. (Zhejiang, China). Myelin oligodendrocyte glycoprotein peptide (MOG) _35–55_ (MEVGWYRSPFSRVVHLYRNGK; purity was >95%) was synthesized by Beijing Xuheyuan Biotech Co., Ltd. (Beijing, China). Complete Freund’ adjuvant (CFA) and pertussis toxin (PTX) were purchased by Sigma-Aldrich (St. Louis, MO, USA). Enzyme-linked immunosorbent assay (ELISA) kits for mouse IL-17A was purchased from eBioScience (San Diego, CA, USA), mouse IL-23 was from Beijing 4A Biotech Co., Ltd. (Beijing, China), mouse IL-6 and mouse TGF-β1 were provided by NeoBioscience Technology Co. (Beijing, China). Mouse anti-IL-17A PERCP-CY5.5, mouse anti-CD4 APC, mouse anti-CD25 FITC, anti-mouse/rat FoxP3 PE, rat IgG isotypecontrol PE, FoxP3 staining buffer set, phorbol 12-myristate 13-acetate (PMA), ionomycin, and brefeldin A solution were purchased from BD PharMingen (San Diego, CA, USA). Rabbit anti-mouse IL-17A and rabbit anti-mouse FoxP3 were purchased from Abcam (Cambridge, UK) and Cell Signaling Technology (Boston, USA), respectively. Rabbit polyclonal anti-β-tubulin was purchased from Epitomics (Burlingame, USA). Western blot (WB) kits were purchased from Applygen Technologies Inc. (Beijing, China). Real-time quantitative reverse transcription PCR (qRT-PCR) kits and reverse transcription kits were purchased from Tiangen Biotech Co., Ltd. (Beijing, China). PCR primers were synthesized by TaKaRa Biotechnology Co. Ltd (Dalian, China).

### Preparation of BSYSC

BSYSC was produced by Beijing Ya Dong Biological Pharmacy Co., Ltd. (Beijing, China). Validation specimens were deposited at the Brain Disease Laboratory of the School of Traditional Chinese Medicine, Capital Medical University, China. BSYSC was composed of *Rehmanniae radix praeparata*, *Radix Rehmanniae*, *Radix Polygoni Multiflori, Radix et Rhizoma Rhei*, *Leonurus japonicas Houtt., Bulbus Fritillariae Thunbergii*, *Hirudo*, *Scorpio*, *Rhiazoma Gastrodiae*, *Fructus Forsythiae*. The proportions of these herbs were 10:10:10:2:10:6:3:2:3:6. *Bulbus Fritillariae Thunbergii* ground into fine powders. The other nine Chinese herbs were extracted twice with boiling water (2 h per extraction, with a total 18-fold volume of purified water). The solutions were concentrated under reduced pressure at 70°C into powders, which were mixed with powders of *Bulbus Fritillariae Thunbergii*. The resulting mixture of powders was encapsulated. To ensure the quality and stability of BSYSC, the active ingredients were identified by ultra-performance liquid chromatography-quadrupole-time-of-flight-mass spectrometry (UPLC-QTOF-MS).

### Identification of active ingredients in BSYSC by UPLC-QTOF-MS

**Figure 1:**
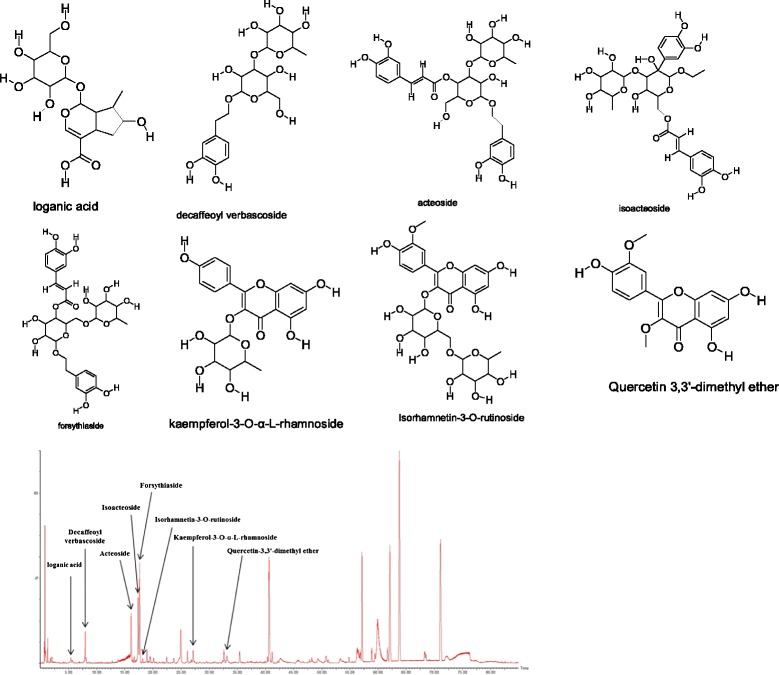
**Chemical structures of active compounds and UPLC-QTOF-MS chromatogram of BSYSC.**

Analysis of the extract was performed by UPLC-QTOF-MS (SYNAPT G2-S, Waters, UK) with a Waters HSS T3 UPLC C18 column (2.1 mm × 150 mm, internal diameter 1.7 μm) set at 35°C. The mobile phase was water (0.1% formic acid, phase A) and acetonitrile (phase B) with a gradient program as follows: 0.0–10.0 min, 95%–95% A; 10.0–15.0 min, 95%–90% A; 15.0–23.0 min, 90%–84% A; 23.0–38.0 min, 84%–60% A; 38.0–63.0 min, 60%–20%; 63.0–85.0 min, 20%–95% A. The flow rate was 0.3 mL/min. The injection volume was 3 μL. The MS spectra were acquired in positive ion mode with an electrospray ionization source (ESI-). The ESI source conditions were: capillary voltage, 2.50 kV; cone voltage, 40 V; quadrupole voltage, 6.0 Bar; ion source temperature, 110°C. Full-scan spectra were acquired in the mass range m/z 100–1,500 m/z. The compounds identified were loganic acid, decaffeoyl verbascoside, acteoside, isoacteoside, forsythiaside, kaempferol-3-O-α-L-rhamnoside, isorhamnetin-3-O-rutinoside and quercetin-3,3’-dimethyl ether were shown in Figure 1, Table 1, respectively.

**Table 1:** **Components of BSYSC identified by UPLC-QTOF-MS**

**Peak no.**	**Retention time (min)**	**Selected ion**	**Formula**	**Compound**	**Measured mass (m/z)**	**Source of drugs**
1	5.42	[M-H]^+^	C_16_H_24_O_10_	loganic acid	375.1132	*Radix Rehmanniae*
2	7.96	[M-H]^+^	C_20_H_30_O_12_	Decaffeoylverbascoside	461.1571	*Radix Rehmanniae*
3	16.14	[M-H]^+^	C_29_H_36_O_15_	Acteoside	623.2045	*Radix Rehmanniae*
4	17.37	[M-H]^+^	C_29_H_36_O_15_	Isoacteoside	623.2043	*Radix Rehmanniae*
5	17.64	[M-H]^+^	C_29_H_36_O_15_	Forsythiaside	623.2072	*Radix Rehmanniae*
6	27.14	[M-H]^+^	C_21_H_20_O_10_	Kaempferol-3-O-α-L-rhamnoside	431.0854	*Radix Polygoni Multiflori*
7	18.16	[M-H]^+^	C_28_H_32_O_16_	Isorhamnetin-3-O-rutinoside	623.2025	*Fructus Forsythiae Suspensae*
8	33.14	[M-H]^+^	C_17_H_14_O_7_	Quercetin-3,3’-dimethyl ether	329.2130	*Fructus Forsythiae Suspensae*

### Quantification of Acteoside and Forsythiasidein in BSYSC

The powder was ultrasonicated (300 W, 40 kHz) in water for 30 min followed by C18 column chromatography. The samples were analyzed by UPLC-QTOF-MS. The contents of the chief components in BSYSC, acteoside and forsythiaside (derived from *Radix Rehmanniae*), were 0.79 mg/g and 0.63 mg/g, respectively.

### Experimental animals

Female C57BL/6 mice (aged 6–8 weeks; weight 16.0-18.0 g) were purchased from Beijing Vital River Laboratories, China [certification NO. SCXK (JING) 2006–0009]. The mice were feed in the Center of Laboratory Animals at Capital Medical University [certification NO. SYXK (JING) 2010–0020]. The mice were housed under a 12-hour light/dark cycle in individual ventilated cages and maintained in a specific pathogen-free grade environment. The experiments were approved by the Ethics Committee of Capital Medical University (No. 2011-X-001).

### Model establishment and experimental treatment

The mice were randomly divided into four groups: normal control (NC, n = 25), EAE model (EAE, n = 25), PA-treated (n = 25) and BSYSC-treated (n = 25). EAE mice were injected subcutaneously (s.c.) with 0.2 mL emulsion containing 50 μg MOG_35–55_ in 100 μL of CFA and 100 μL of normal saline (NS), followed by intraperitoneal (i.p.) injections of 500 ng of PTX on Day 0 and Day 2 post-induction (PI) [16]. The 3.02 g/kg dose of BSYSC was shown to be more effective against EAE in our previous study [17]. In the study, the mice in the BSYSC-treated EAE group were given oral suspensions of 3.02 g/kg BSYSC once a day for 40 days. The PA-treated EAE mice were administered PA at a dose of 6 mg/kg. NC and EAE mice were treated with NS. Neurological function scores were based on the sum of the disease state for the tail and all four limbs as follows: 0, no signs of disease in the tail and limbs; scores for the tail were assigned as 1 for paralyzed tail tipparalysis and 2 for tail flaccidity; scores for the limbs were assigned as 1 for gait disturbance, 2 for moderate paralysis and limb dragging and 3 for total paralysis. Mortality was assigned a score of 15 [18]. The grip test records down time from glass bar, which wrapped round gauze to avoid slip of mice. The grip strength test scores were defined as: 0 for 0–4 s, 1 for 5–9 s, 2 for 10–14 s, 3 for 15–19 s, 4 for 20–24 s, 5 for 25–29 s, and 6 for more than 30 s [19].

### Sample collection

The mice were sacrificed on Day 6 (early stage, no neurological signs), Day 18 (acute stage, neurological function scores at a peak) and Day 40 (remission stage, no further increase in the signs of EAE) PI. The spleen of four mice in each group were removed and prepared for flow cytometric (FCM) analysis; the brain and spinal cord were immediately frozen for ELISA, qRT-PCR and WB analyses; and 4% paraformaldehyde was used to fix the brain and spinal cord for hematoxylin-eosin (H&E) staining, transmission electron microscopy (TEM) and immunohistochemical (IHC) analysis.

### Histopathology and ultrastructural pathology

The brain and spinal cord were fixed, embedded in paraffin, sectioned (thickness, 3 μm) for H&E staining. The sections were observed with light microscopy (Nikon Eclipse 80i, Tokyo, Japan). The inflammatory cell infiltration was scored as follows: 0, no infiltrate; 1, scattered inflammatory cells; 2, single inflammatory cells around blood vessels; 3, inflammatory cell infiltration surrounding blood vessels; 4, inflammatory cell infiltration and perivascular cuff formation, or parenchymal necrosis [20,21]. Cross-sections (1 mm) of the intumescentia lumbalis of the brain and spinal cord were embedded in Epon and sectioned on a Leica EM ultramicrotome. Thin sections (thickness, 70 nm) were viewed on a TEM (JEM-1230, Japan) using a digital camera system to obtain micrographs. Ten high-power fields (×10,000) were selected in the brain (white matter) and spinal cords in each group. The images were analyzed by Image-Pro Plus 6.0 (Media Cybernetics, Inc., Washington, USA) and the results were expressed as area/diameter (mean) of myelin [22-24].

### Intracellular cytokine analysis by FCM

A single cell suspension of spleen mononuclear cells was first permeabilized with fixation/permeabilzation solution for 30 min at 4°C, washed in perm/wash buffer, and then incubated with mouse anti-CD4 APC, mouse anti-CD25 FITC and mouse anti-FoxP3 PE for 60 min at 4°C. Alternatively, spleen mononuclear cells were incubated with mouse anti-CD4 APC, mouse anti-CD25 FITC and mouse anti-IL-17A PERCP-CY5.5 for 5 hours at 37°C. A control group treated with isotype control antibody was prepared. Cells were fixed in formaldehyde and then analyzed on a BD-FACS Calibur (USA). Data were analyzed based on the percentages of Th17 and Treg cells.

### Cytokine measurements by ELISA

The brain of each mouse was weighed and homogenized with cold NS and centrifuged at 3,000 rpm for 20 min to obtain supernatants for cytokine ELISA conducted in strict accordance with instructions provided by the manufacturers. Cytokine (IL-17A, IL-6, IL-23 and TGF-β1) concentrations were determined using the relevant standard curves.

### mRNA Analysis by qRT-PCR

Total RNA was isolated from approximately 30 mg brain or spinal cord of mice according to the manufacturer’s instructions. RNA samples with an OD_260_/OD_280_ ratio of 1.9-2.1 and an OD_260_/OD_230_ ratio greater than 2.0 were used for the analysis. The cDNA was synthesized from total RNA by the reverse transcription of 1 μg of total RNA using the reverse transcription kit. The primer sequences for PCR designed by the primer premier 5.0 software, based on the GenBank sequences were as follows: FoxP3 (NM_001199347.1) Forward (F), 5’-CTGCCTTGGTACATTCGTGAAC-3’, FoxP3 Reverse (R), 5’-ATGTTGTGGGTGAGTGCTTTG-3’; IL-17A (NM_010552.3) F, 5’-GGCTGACCCCTAAGAAACC-3’, IL-17A R, 5’-CTGAAAATCAATAGCACGAAC-3’; β-actin (NM_007393.3) F, 5’-CTGAAAATCAATAGCACGAAC-3’ and β-actin R, 5’-ATGGAGCCACCGATCCACA -3’. The amplified fragments were 97, 79 and 171 base pairs (bp), respectively. The PCRs were carried out using the following conditions: 95°C for 15 min, followed by 40 cycles of denaturation at 95°C for 10 s, annealing at 52°C for 30 s and extension at 72°C for 31 s (Applied Biosystems 7300, Foster, USA). The relative quantification (RQ) was analyzed by the 2^-ΔΔCt^ method.

### Western blot analysis

Protein extraction and quantification were performed according to the procedures specified by the manufacturers of the reagents used. Each sample containing 20 μg protein was separated by 5% and 10% SDS-PAGE and electrotransferred onto polyvinylidene fluoride membranes (Millipore, USA). The membranes were incubated with primarily anti-IL-17A antibody (1:1,000), anti-FoxP3 antibody (1:2,000), and rabbit polyclonal anti-β-tubulin antibody (1:50,000) in blocking solution at 4°C overnight. Then, the membranes were incubated with secondary goat anti-rabbit IgG (1:20,000) for 60 min and electrochemiluminescence (ECL) reagent for 1 min, followed by exposure to Kodak film (Japan). Data were represented by the integrated optical density (IOD) ratio determined using analysis software ImageQuant TL 2005 image analysis software (Amersham, Biosciences, Piscataway, NJ).

### Immunohistochemical analysis

The sections were incubated with primary detection antibody [rabbit anti-mouse IL-17A (Abcam, Cambridge, UK, antibody concentration, 1:100), rabbit anti-mouse FoxP3 (Abcam, Cambridge, UK, antibody concentration, 1:80)] at 4°C for 40 h. Subsequently, the sections were incubated with the biotin-labeled secondary antibody (sheep anti-rabbit IgG) at 37°C for 60 min. Color development was accomplished by exposure to 3,3’-diaminobenzidinetetrahydrochloride (DAB) for 40 seconds to 1 min. Finally, the sections were dehydrated and mounted for microscopic observation. Quantitative analysis of the immunohistochemically stained images was carried out with a NIS-Elements BR 3.0 system. Three to five high-power fields (×400) were selected from four sections of the lateral ventricle in each group and positive results were expressed as IOD values.

### Statistical analysis

The data were expressed as mean ± standard error (SE) and analyzed with SPSS version 17.0 (SPSS Inc., Chicago, IL, USA). All the data were firstly subjected to descriptive statistics for normality. The data with normally distributed and equal variances were examined using one-way ANOVA with a post-hoc LSD test, otherwise, the data were performed with a rank-sum test. The family-wise error rate was controlled by the statistical method of Bonferroni. A value of *p* < 0.05 was considered to indicate statistical significance.

## Results

### Neurological function and grip test scores

**Figure 2:**
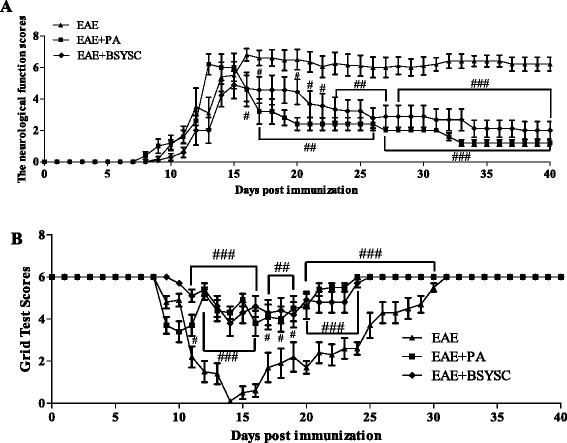
**The neurological function scores (A) and the grip strength test scores (B) of mice.**

Symptoms of EAE including flaccid tail, staggering gait, hind-limb paralysis, four-limb paralysis and even death appeared sequentially in experimental mice from Day 8 PI. The neurological function scores of EAE mice reached a peak on Day 16. These scores were decreased significantly on Day 17 PI, and from Day 20 to Day 40 PI in BSYSC-treated mice, compared to the scores in EAE mice (*p* < 0.05, *p* < 0.01, *p* < 0.001, respectively; Figure 2). The grip strength test scores of EAE mice appeared primarily from Day 9 PI, with the falling time for EAE mice reduced to less than 30 seconds and the scores reaching a nadir on Day 14 PI. Compared to EAE mice, the scores were significantly higher in BSYSC-treated EAE mice from Day 11 to Day 30 PI (*p* < 0.01 or *p* < 0.001, respectively; Figure 2).

### Pathological changes on brain and spinal cord tissues

**Figure 3:**
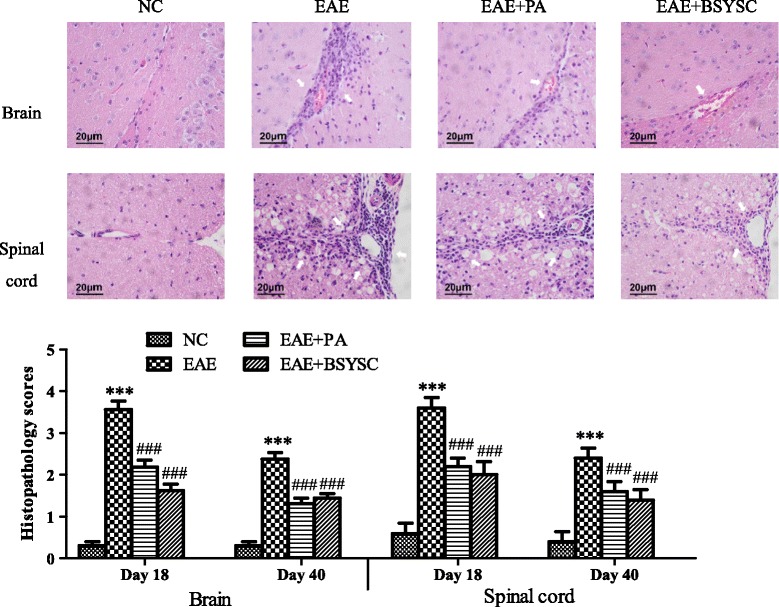
**Observation of pathological changes in the brain and spinal cord of mice under the light microscope (scale bar, 20 μm) on Day 18 PI of EAE.** NC mice showed normal structures, while EAE mice had large amounts of inflammatory cell infiltration. PA- and BSYSC-treated EAE mice exhibited a small number of inflammatory cells than EAE mice. Note: ^***^ *p* <0.001 vs.NC; ^###^ *p* <0.001 vs. EAE.

**Figure 4:**
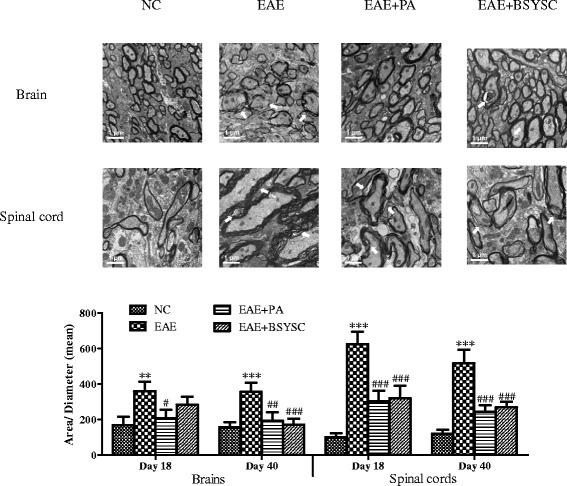
**Pathological changes visualized under TEM on Day 18 PI (scale bar 0.5 μm).** NC mice showed normal structures, while EAE mice had large amounts of demyelination. PA- and BSYSC-treated EAE mice exhibited a lighter demyelination than EAE mice. The results were expressed as a total area of / diameter (mean) of myelin. Note: ^**^ *p* <0.01, ^***^ *p* <0.001 VS NC; ^#^ *p* <0.05, ^##^ *p* <0.01, ^###^ *p* <0.001 VS EAE.

The pathological changes in the brain and spinal cord of mice were observed by light microscopic evaluation of H&E stained tissues on Day 18 and Day 40 PI. The neuronal structure was normal in the NC group. A large number of inflammatory cells were aggregated around small blood vessels to form “sleeve-like” structures in the EAE model group. Conversely, treatment with PA or BSYSC reduced inflammatory cell infiltration. The type of inflammatory cells was classified by lymphocytes, monocytes, neutrophils and eosinophils. The histopathology scores of EAE mice were dramatically increased compared to those of NC mice on Day 18 and Day 40 PI, while treatment with PA or BSYSC significantly reduced the scores (*p* < 0.001; Figure 3). In further studies, the ultrastructure of the myelin sheath and axons in the brain and spinal cord was observed by TEM. While these were found to be normal in NC mice, a fluffy layer, axonal edema and disintegration were observed in the EAE mice. Demyelination and axonal damage was ameliorated in the PA- and BSYSC-treated EAE mice. The damage was quantified with the ratio of area/diameter (mean) of myelin. The results showed that the ratios of in the brain and spinal cord of EAE mice were markedly increased compared to those of NC mice on Day 18 and 40 PI (*p* < 0.05 or *p* < 0.001, respectively; Figure 4). The treatment with PA or BSYSC significantly reduced the ratios (*p* < 0.05, *p* < 0.01, *p* < 0.001, respectively; Figure 4).

### FCM analysis of splenic CD4 + IL-17+ and CD4 + CD25 + FoxP3+ T cells

**Figure 5:**
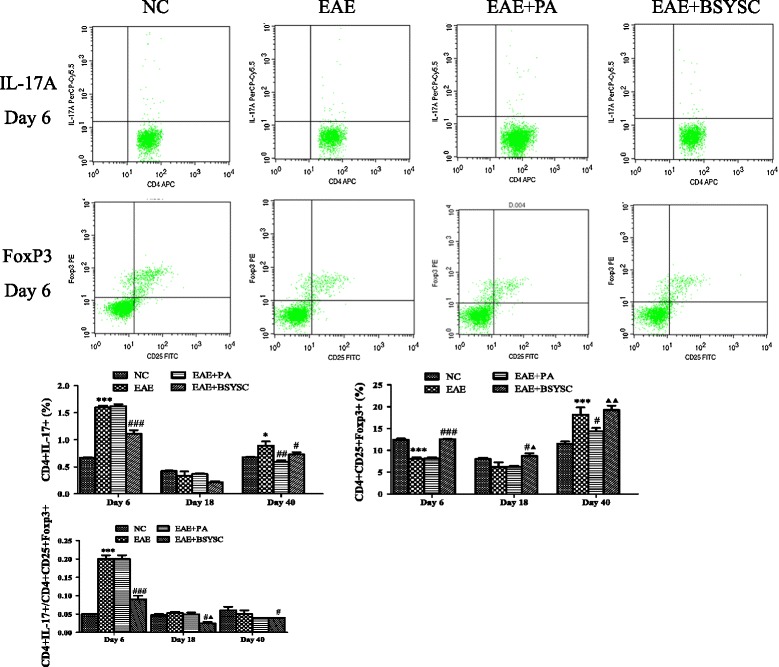
**Effect of BSYSC on the expressions of CD4 + IL-17+ T cells and CD4 + CD25 + FoxP3+ T cells in the spleen of mice.** It was assessed by FCM. Note: ^*^ *p* <0.05, ^**^ *p* <0.01, ^***^ *p* <0.001 VS NC;^#^ *p* <0.05, ^##^ *p* <0.01, ^###^ *p* <0.001 VS EAE; ^▲^ *p* <0.05, ^▲▲^ *p* <0.01 VS EAE + PA.

To assess the changes in splenic Th17 and Treg cells in EAE mice, the levels of CD4 + IL-17+ and CD4 + CD25 + FoxP3+ T cells were measured by FCM. The ratios of CD4 + IL-17+/CD4 + CD25 + FoxP3+ were also analyzed. There was a significant increase in CD4 + IL-17+ cells in the EAE mice, compared to the NC mice on Day 6 and Day 40 PI (*p* < 0.001 and *p* < 0.05, respectively). There was a significant decrease in CD4 + CD25 + FoxP3+ cells on Day 6 PI (*p* < 0.001), but the levels were increased on Day 40 PI (*p* < 0.001). The CD4 + IL-17+/CD4 + CD25 + FoxP3+ ratio was markedly increased only on Day 6 PI (*p* < 0.001). BSYSC obviously decreased CD4 + IL-17+ levels on Day 6 and Day 40 PI compared to EAE mice (*p* < 0.001 and *p* < 0.05, respectively). PA had similar effects to BSYSC in lowering the CD4 + IL-17+ levels on Day 40 PI (*p* < 0.01). BSYSC obviously increased CD4 + CD25 + FoxP3+ T cell levels compared to EAE mice on Day 6 and Day 18 PI (*p* < 0.001 and *p* < 0.05, respectively). The CD4 + IL-17+/CD4 + CD25 + FoxP3+ ratio was significantly decreased by BSYSC treatment during EAE induction on Days 6, 18 and 40 PI (*p* < 0.001, *p* < 0.05, Figure 5).

### ELISA analysis of inflammation-related cytokines in brain tissue

**Figure 6:**
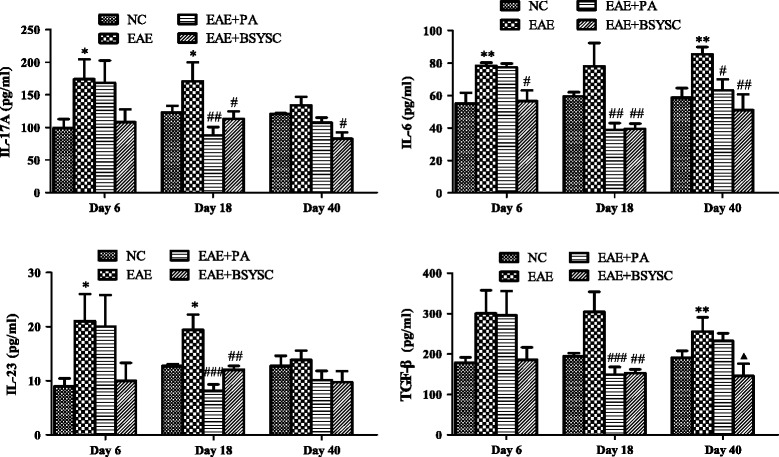
**ELISA analysis of IL-17A, IL-6, IL-23 and TGF-β1 expressions in the brain of the groups.** Note:^*^ *p* <0.05, ^**^ *p* <0.01 vs. NC; ^#^ *p* <0.05, ^##^ *p* <0.01, ^###^ *p* <0.001 vs. EAE; ^▲^ *p* <0.05 vs. EAE + PA.

Compared to NC mice, the levels of IL-17A, IL-6, IL-23 and TGF-β1 in the brain of EAE mice were significantly increased at various times PI: IL-17A and IL-23 levels on Days 6 and 18 PI (*p* < 0.05), IL-6 on Days 6 and 40 PI (*p* < 0.01), and TGF-β1 on Day 40 PI (*p* < 0.01). In contrast, the levels of these cytokines decreased in EAE mice treated with PA or BSYSC. Compared to EAE mice, IL-17A was significantly decreased in PA-treated EAE mice on Day 18 PI (*p* < 0.01), and in BSYSC-treated EAE mice on Days 18 and 40 PI (*p* < 0.05). IL-6 was decreased in PA-treated EAE mice on Days 18 and 40, and in BSYSC-treated EAE mice on Days 6, 18 and 40 PI (*p* < 0.05 and *p* < 0.01). IL-23 and TGF-β1 were decreased in PA- and BSYSC-treated EAE mice on Day 18 PI (*p* < 0.001 and *p* < 0.01, respectively; Figure 6).

### qRT-PCR analysis of IL-17A and FoxP3 mRNA expression in brain and spinal cord tissue

**Figure 7:**
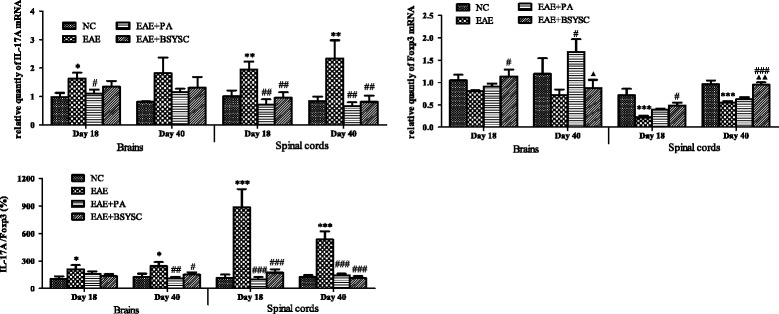
**qRT-PCR analysis of the effect of BSYSC on mRNA expressions of IL-17A and FoxP3 in the brain and spinal cord of mice.** Note: ^*^ *p* <0.05, ^**^ *p* <0.01, ^***^ *p* <0.001 vs. NC; ^#^ *p* <0.05, ^##^ *p* <0.01, ^###^ *p* <0.001 vs. EAE; ^▲^ *p* <0.05, ^▲▲^ *p* <0.01 vs. EAE + PA.

Compared to NC mice, IL-17A mRNA levels were significantly increased in the brain on Day 18 PI (but not at Day 40) and in the spinal cord on Days 18 and 40 PI in EAE mice compared to NC mice (*p* < 0.05, *p* < 0.01). In contrast, the levels were significantly decreased in PA- and BSYSC-treated EAE mice compared to EAE mice in the spinal cord (*p* < 0.01). FoxP3 mRNA in the spinal cord was significantly decreased in EAE mice compared to NC mice (*p* < 0.001), however, there were no differences in the levels detected in the brain of the two groups. BSYSC increased FoxP3 mRNA levels in the spinal cord of EAE mice on Days 18 and 40 PI (*p* < 0.05 and *p* < 0.001, respectively). Analysis of the ratio of IL-17A/FoxP3 mRNA levels revealed a significant increase in both the brain and spinal cord on Days 18 and 40 PI in EAE mice compared to NC mice (*p* < 0.05 and *p* < 0.001, respectively). In contrast, the ratio was significantly decreased in spinal cord on Days 18 and 40 PI and in the brain on Day 40 PI in PA- and BSYSC-treated EAE mice compared to EAE mice (Figure 7).

### WB and IHC analysis of IL-17A and FoxP3 proteins in brain and spinal cord tissue

**Figure 8:**
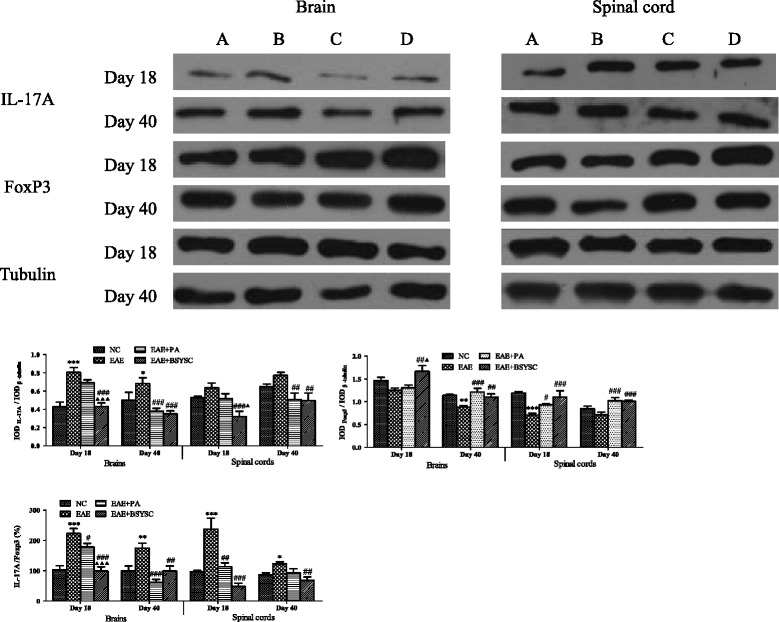
**Western blot analysis of the effect of BSYSC on IL-17A and FoxP3 protein expressions in the brain and spinal cord of mice.** (A) to (D) NC, EAE, EAE + PA and EAE + BSYSC mice. Note: ^*^ *p* <0.05, ^**^ *p* <0.01, ^***^ *p* <0.001 vs. NC; ^#^ *p* <0.05, ^##^ *p* <0.01, ^###^ *p* <0.001 vs. EAE; ^▲^ *p* <0.05, ^▲▲▲^ *p* <0.001 vs. EAE + PA.

**Figure 9:**
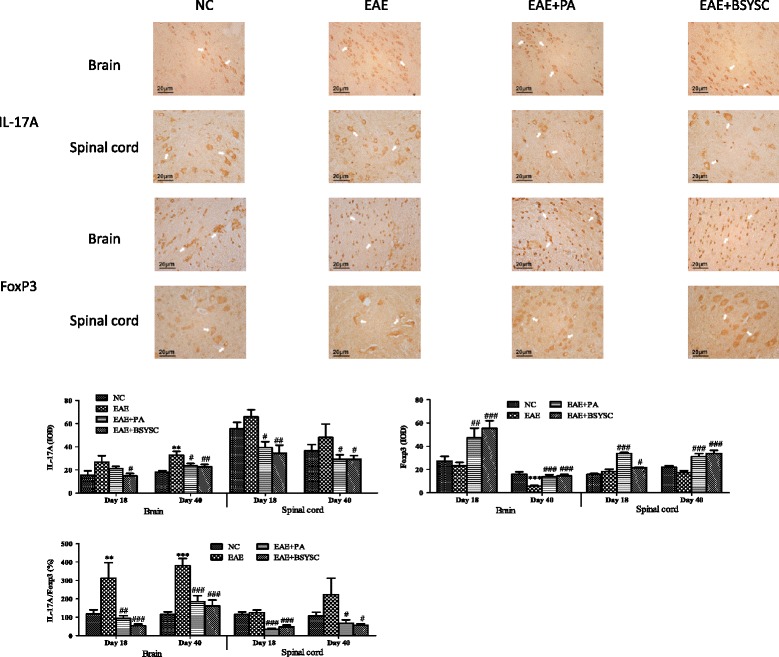
**IHC analysis of the effect of BSYSC on IL-17A and FoxP3 protein expressions in the brain and spinal cord of mice.** Note: ^**^ *p* <0.01, ^***^ *p* <0.001 vs. NC; ^#^ *p* <0.05, ^##^ *p* <0.01, ^###^ *p* <0.001 vs. EAE.

WB analysis demonstrated that IL-17A protein expression in the brain was significantly increased in EAE mice compared to NC mice on Days 18 and 40 PI (*p* < 0.001 and *p* < 0.05, respectively). In contrast, levels were significantly reduced in the brain and spinal cord on Days 18 and 40 PI in BSYSC-treated EAE mice compared to EAE mice (*p* < 0.001 and *p* < 0.01, respectively). In addition, compared to NC mice, FoxP3 protein expression was obviously decreased in the brain of EAE mice on Day 40 PI and in the spinal cord on Day 18 PI (*p* < 0.01 and *p* < 0.001, respectively). BSYSC significantly increased FoxP3 protein in the brain and spinal cord of EAE mice on Days 18 and 40 PI (*p* < 0.01 and *p* < 0.001, respectively). Furthermore, the IL-17A/FoxP3 ratios were significantly increased in the brain and spinal cord of EAE mice on Days 18 and 40 PI compared to NC mice (*p* < 0.001 and *p* < 0.01 and *p* < 0.05, respectively). In contrast, the ratios were significantly reduced in the brain and spinal cord of BSYSC-treated EAE mice, compared to EAE mice on Days 18 and 40 PI (*p* < 0.001 and *p* < 0.01, respectively; Figure 8). IL-17A and FoxP3 protein expressions in the brain and spinal cord were also detected morphologically by IHC. The results showed that the change trends of IL-17A and FoxP3 protein by IHC were similar to WB (Figure 9).

## Discussion

The results of this study showed the EAE induced by administration of MOG_35–55_ was pathologically characterized by mononuclear cells infiltration, cells shrinkage and nucleuses lysogenic, with neurological deficits associating with demyelination occurred in the brain and spinal cord, which are consistent with previous reports [25]. Comparing with EAE, the EAE + BSYSC manifested an improved neurological function, ameliorated inflammation and demyelination in the CNS. Combining with our previous studies [26], these observations suggested that BSYSC had the neuroprotective effects in EAE mice.

To analyze the neuroprotective mechanisms of BSYSC, we first investigated the development of Th17 and Treg responses in the EAE spleen. It is worth noting that the ratio of CD4 + IL-17+ and CD4 + CD25 + FoxP3 + T cells was obviously increased on Day 6 PI in the spleen of EAE mice. It has been reported that the number of IL-17A-secreting PLP-specific CD4+ T cells, measured by enzyme-linked immunospot assay (ELISPOT), increased significantly in the spleen of SJL mice on Day 8 and in the CNS on Day 25 following the induction of EAE by PLP_139–151_ administration [27]. It was indicated that the inflammation reaction was initiated in the peripheral immune organs in the early stage, producing cytokines that disrupt the blood brain barrier (BBB) and allowing immune cells to enter the CNS. Thus, CNS damage occurs as a consequence of peripheral immune responses [28]. Further studies demonstrated that the early treatment with Fasudil, a selective Rho kinase (ROCK) inhibitor, resulted in down-regulation of CD4 + IL-17+ T cells in the spleen on Day 9 PI [29]. In our study, BSYSC significantly regulated the balance between the Th17 and Treg T cells in the spleen of EAE mice in the early stage, resulting in a reduction in the inflammatory responses in peripheral immune organs.

Moreover, we found the levels of inflammation-related cytokines, such as IL-17A, IL-6 and IL-23 increased during the entire course of the disease in the brain of EAE mice. IL-17A, the predominant functional member of the IL-17 family, is a pro-inflammatory cytokine produced by Th17 cells [30]. IL-6 is strongly induced by cells of the innate immune system [31]. Studies have shown that the expressions of these two cytokines are significantly increased in MS/EAE [32,33]. IL-23 is an important factor in Th17 differentiation and the development of EAE [34] and studies have shown that IL-23 and IL-17 secreting cells are attacked to the CNS, leading to demyelination [35,36]. Both PA and BSYSC down regulated the expressions of IL-17A, IL-6 and IL-23 at different stage of EAE, especially in the acute stage.

TGF-β1 has been reported to stimulate naïve CD4+ T cells to differentiate into Treg cells which also produce TGF-β1 [37]. Although TGF-β1 exerts an anti-inflammatory activity via the induction of FoxP3 expression, in combining with IL-6 and IL-23, TGF-β1 stimulates the production of Th17 cells in EAE [38,39]. In the present study, the high TGF-β1 level in the brain can be speculated to the induction of Th17 cell differentiation by the combination of IL-6 and IL-23, resulting in the exacerbation of EAE [40]. This is supported by the observation that BSYSC reduced not only the levels of IL-17A, IL-6 and IL-23, but also of TGF-β1.

Finally, we targeted the regulatory role of BSYSC played on the IL-17A/FoxP3 ratio in the brain and spinal cord in EAE mice. IL-17A acts an encephalitogenic part in the EAE model, with accumulation of Th17 cells and elevation of IL-17A levels observed in the brain and spinal cord of EAE mice [41,42]. Furthermore, mice lacking Treg cells but having MBP-specific effector T cells can develop spontaneous EAE [43]. In previous literature, immune dysfunction is related with the up-regulation of Th17 cells and down-regulation of the Treg cells [40,44]. Therefore, the regulation of immunity balance between Th17 and Treg cells is implicated as novel drug targets for the treatment of MS. Recently, the neuroprotective effect against EAE with electroacupuncture (EA) was reported, the regulation of Th1/Th2/Th17/Treg by EA was observed as well [45]. However, few studies have investigated the effects of formulas in different tissues and during different stages of EAE. In this study, we found that BSYSC regulated the balance between IL-17A and FoxP3 mRNA and protein expression in the CNS of EAE mice.

Meanwhile, the 8 chemical components of BSYSC were identified. Several studies indicates thatiridoids (loganic acid), phenylpropanoids (decaffeoyl verbascoside, acteoside, isoacteoside and forsythiaside), flavonoids (kaempferol-3-O-α-L-rhamnoside and Isorhamnetin-3-O-rutinoside) possess anti-inflammatory, anti-oxidative, immune-regulating and neuroprotective effects [46-52]. Additionally, some of the herbs in BSYSC, such as *Radix Rehmanniae*, *Radix Polygoni Multiflori* and *Fructus Forsythiae Suspensae* also have the above effects [53-59]. These findings implied the effects of BSYSC in MS/EAE were considered to be related to the entire interactive action of multiple components and herbs.

## Conclusion

In this study, BSYSC exerts significant neuroprotective effects against EAE in mice. The mechanism is related to the decreased number of lymphocytes in the peripheral inflammatory system, thereby reducing the entry of cytotoxic T cells into CNS, and mediating regulation of Th17 and Treg cells and the related cytokines in the CNS. This study provides a basis for further research into the efficacy of BSYSC and its active ingredients in the treatment of MS. However, the formula contained within BSYSC is complicated and the components exert multi-target effects, thus, the other mechanisms by which BSYSC influences disease in EAE mice remain poorly understood and require further investigation.

## Abbreviations

BSYSC: Bu Shen Yi Sui Capsule (a traditional Chinese medicine)
MS: Multiple sclerosis
EAE: Experimental autoimmune encephalomyelitis
TGF: Transforming growth factor
ROR: Retinoic acid-related orphan receptor
FoxP3: Forkhead box P3
PA: Prednisone acetate
TCM: Traditional Chinese Medicine
MOG: Myelin oligodendrocyte glycoprotein peptide
CFA: Complete Freund’ adjuvant
PTX: Pertussis toxin
PMA: Phorbol 12-myristate 13-acetate
qRT-PCR: Real-time quantitative reverse transcription PCR
UPLC-QTOF-MS: Ultra-performance liquid chromatography-quadrupole-time-of-flight-mass spectrometry
ELISPOT: Enzyme-linked immunospot assay
ROCK: Rho kinase
EA: Electroacupuncture
